# Decreased autophagy induced by β_1_-adrenoceptor autoantibodies contributes to cardiomyocyte apoptosis

**DOI:** 10.1038/s41419-018-0445-9

**Published:** 2018-03-14

**Authors:** Li Wang, Yang Li, Na Ning, Jin Wang, Zi Yan, Suli Zhang, Xiangying Jiao, Xiaohui Wang, Huirong Liu

**Affiliations:** 1 Department of Pathology, Shanxi Medical University, 030001 Taiyuan, China; 2 Department of Physiology, Shanxi Medical University, 030001 Taiyuan, China; 3 Department of Physiology and Pathophysiology, School of Basic Medical Sciences, Capital Medical University, 100069 Beijing, China

## Abstract

It has been recognized that myocardial apoptosis is one major factor in the development of heart dysfunction and autophagy has been shown to influence the apoptosis. In previous studies, we reported that anti-β_1_-adrenergic receptor autoantibodies (β_1_-AABs) decreased myocardial autophagy, but the role of decreased autophagy in cardiomyocyte apoptosis remains unclear. In the present study, we used a β_1_-AAB-immunized rat model to investigate the role of decreased autophagy in cardiomyocyte apoptosis. We reported that the level of autophagic flux increased early and then decreased in an actively β_1_-AAB-immunized rat model. Rapamycin, an mTOR inhibitor, restored myocardial apoptosis in the presence of β_1_-AABs. Further, we found that the early increase of autophagy was an adaptive stress response that is possibly unrelated to β_1_-AR, and the activation of the β_1_-AR and PKA contributed to late decreased autophagy. Then, after upregulating or inhibiting autophagy with rapamycin, Atg5 overexpression adenovirus or 3-methyladenine in cultured primary neonatal rat cardiomyocytes, we found that autophagy decline promoted myocardial apoptosis effectively through the mitochondrial apoptotic pathway. In conclusion, the reduction of apoptosis through the proper regulation of autophagy may be important for treating patients with β_1_-AAB-positive heart dysfunction.

## Introduction

Cardiac dysfunction is one of the most common causes of cardiovascular disease^1^, however, its pathogenesis has not been fully elucidated. Apoptosis plays a pivotal role in the occurrence and development of cardiac dysfunction; both animal experiments and human studies have found that cardiomyocyte apoptosis occurs in the deterioration of cardiac function, and the inhibition of apoptosis could effectively attenuate cardiac dysfunction^2^. Therefore, the effective reduction of myocardial apoptosis is important in the prevention and treatment of heart dysfunction.

There are indications that β_1_-adrenoceptor autoantibodies (β_1_-AABs) can be detected in the serum of 40–60% of patients with cardiac dysfunction^3^. Studies have shown that β_1_-AABs could induce cardiomyocyte apoptosis through the β_1_-adrenergic receptor (β_1_-AR)^4^, which is followed by the deterioration of cardiac function. However, it is still unclear how β_1_-AABs cause apoptosis of cardiac myocytes.

Autophagy, which is an important mechanism of maintaining cellular homeostasis, has been shown to influence the apoptosis^5^. Impaired organelles or incorrectly folded proteins are degraded by autophagy in order to provide a critical means for cell self-renewal, energy repletion, and substrate recycling^6^. In preliminary studies, our group has shown that decreased autophagy induced by β_1_-AABs contributed to cardiomyocyte death and cardiac dysfunction^7^. In certain circumstances, autophagy, as a stress response, can protect cells from death by inhibiting apoptosis^8^, while the inhibition of autophagy by 3-methyladenine (3-MA) or the silencing of Atg5 or Atg7 could activate caspase-3 and subsequently apoptosis^9^. Therefore, autophagy might be essential to the occurrence of apoptosis. However, whether autophagy influences cardiomyocyte apoptosis induced by β_1_-AABs is still unknown.

In the present study, an actively β_1_-AAB-immunized rat model and cultured primary neonatal rat cardiomyocytes were used to observe the possible mechanism of β_1_-AAB-induced apoptosis from the autophagy perspective. The purpose is to show whether the regulation of autophagy may play a therapeutic role in β_1_-AAB-positive patients with heart disease.

## Results

### β_1_-AABs caused apoptosis of myocardial tissues in actively immunized rats

**Fig. 1:**
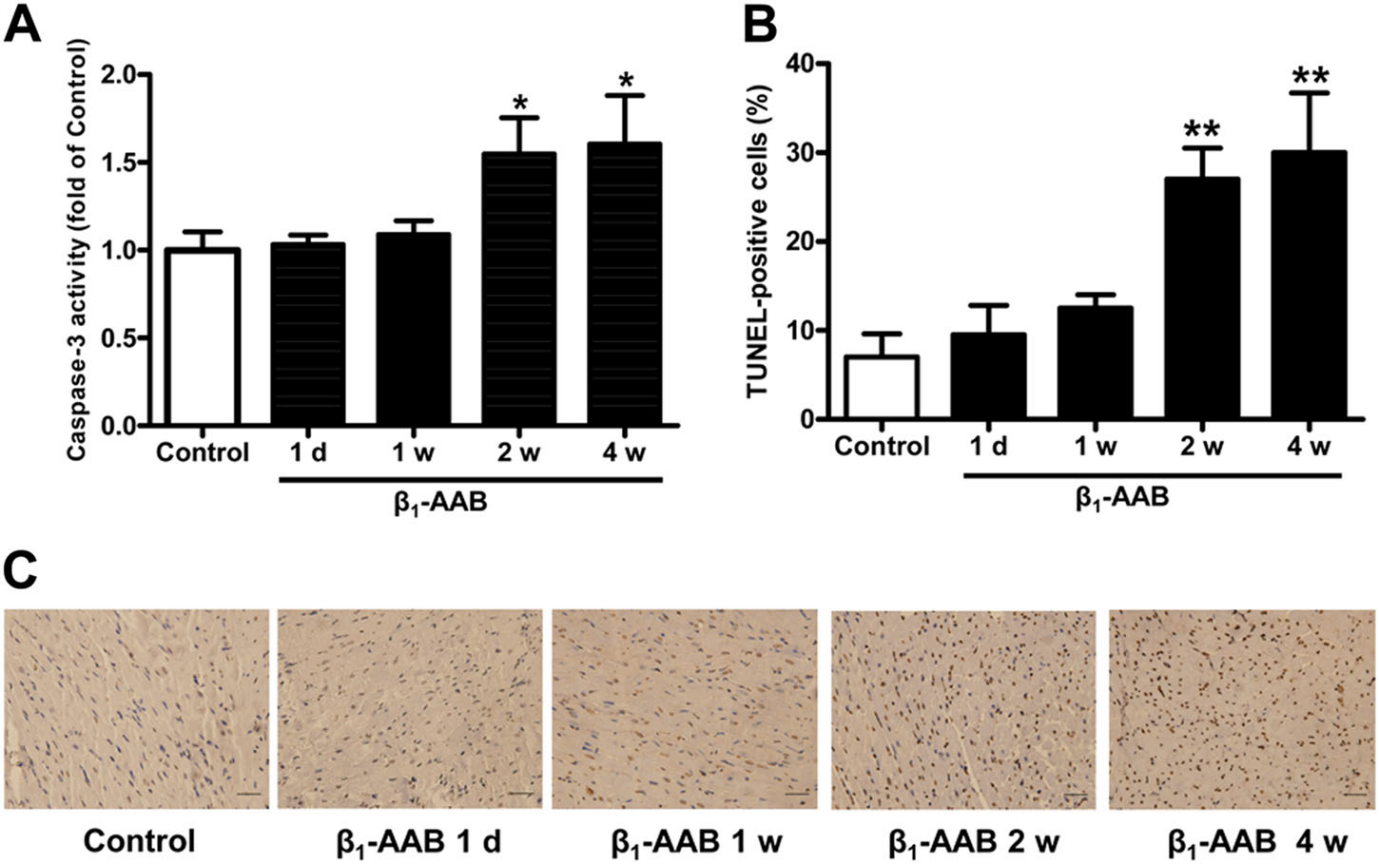
Increased β_1_-AAB-induced apoptosis in myocardial tissues after active immunization. **a** Caspase-3 activity at 0, 1 day, 1 week, 2 weeks, and 4 weeks after active immunization. **b** Quantification of TUNEL-positive cells from (**c**). **c** Representative TUNEL staining of myocardial tissues. Scale bar was 40 μm. Data are expressed as means ± SEM (*n* = 6 per group). *P < 0.05 vs. the control and **P < 0.01 vs. the control

In this study, a caspase-3 activity assay and TUNEL staining were used to detect the apoptosis level of myocardial tissues in actively immunized rats. There was no significant change of caspase-3 activity and the number of TUNEL-positive cells at 1 day and 1 week after active immunization, but they began to increase at 2 weeks and remained at a high level until 4 weeks (Fig. 1). The above results showed that β_1_-AABs could promote apoptosis in myocardial tissues.

### Myocardial autophagic flux increased early and then decreased with the presence of β_1_-AABs

**Fig. 2:**
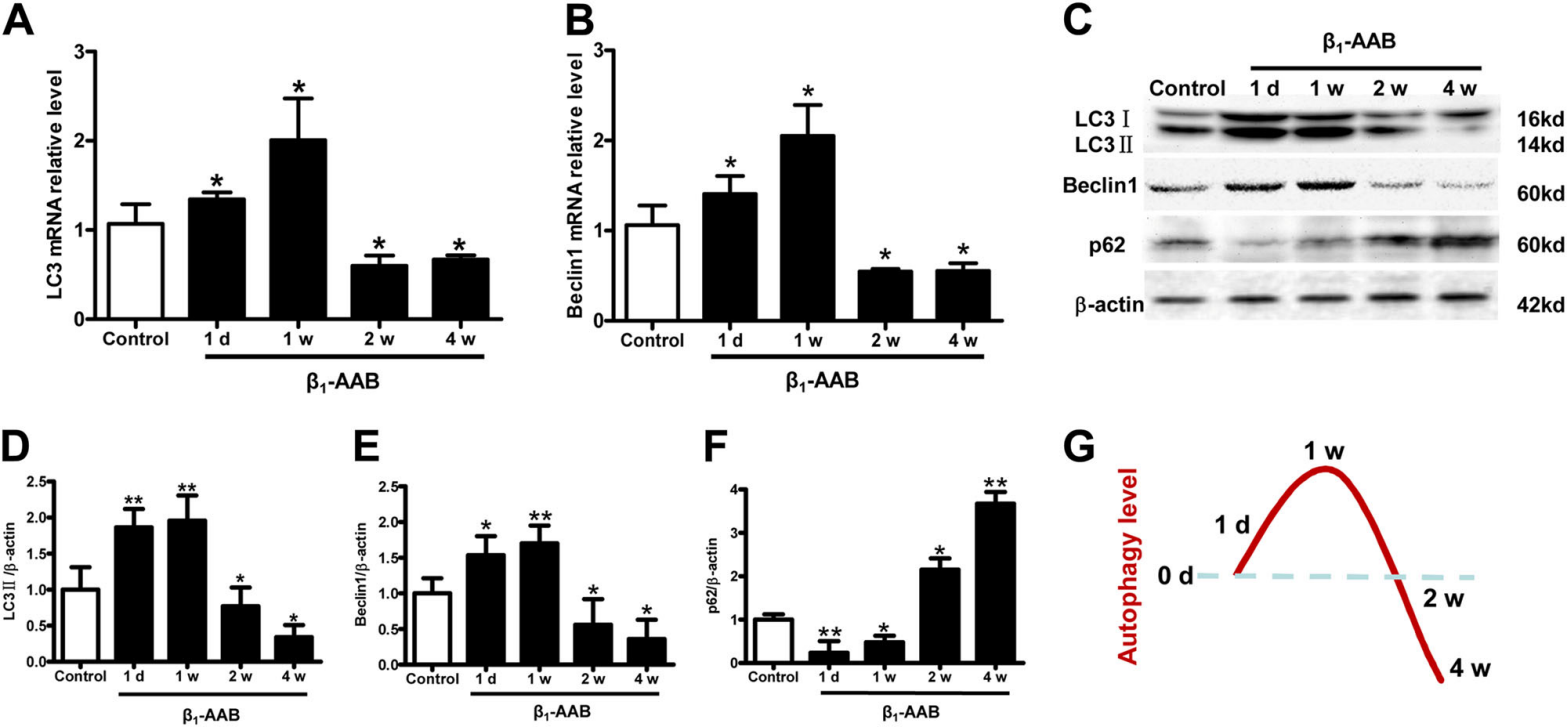
Change in autophagic flux induced by β_1_-AABs in myocardial tissues of actively immunized rats. **a**, **b** Real-time PCR was used to measure LC3 and Beclin1 mRNA expression in cardiomyocytes. **c** Representative Western blot showing the protein expression of LC3, Beclin1, and p62 at 0, 1 day, 1 week, 2 weeks, and 4 weeks after active immunization. **d–f** Quantification of Western blot data from (**c**). **g** Schematic representation of time course of autophagy level in myocardial tissue of the actively β_1_-AAB-immunized rat model. Data are expressed as means ± SEM (*n* = 6 per group). *P < 0.05 vs. the control and **P < 0.01 vs. the control

The expression of LC3 and Beclin1 was detected to reflect the changes of autophagy in myocardial tissues of actively immunized rats. The results revealed that the mRNA and protein levels of LC3 and Beclin1 were significantly increased at 1 day after active immunization; they peaked at 1 week, and then began to decrease at 2 weeks compared with the control group (Fig. 2).

To reflect the variation of the autophagic flux, the selective autophagy substrate p62 was detected. Our results showed that the p62 level was significantly decreased 1 day and 1 week after active immunization. However, p62 accumulated after 2 weeks, and a further p62 increase appeared after 4 weeks (Fig. 2). The results above reminded us that active immunization led to an earlier increase but a later decrease of myocardial autophagy flux in rats.

### Upregulation of autophagy by RAPA reduced cardiomyocyte apoptosis in actively immunized rats

**Fig. 3:**
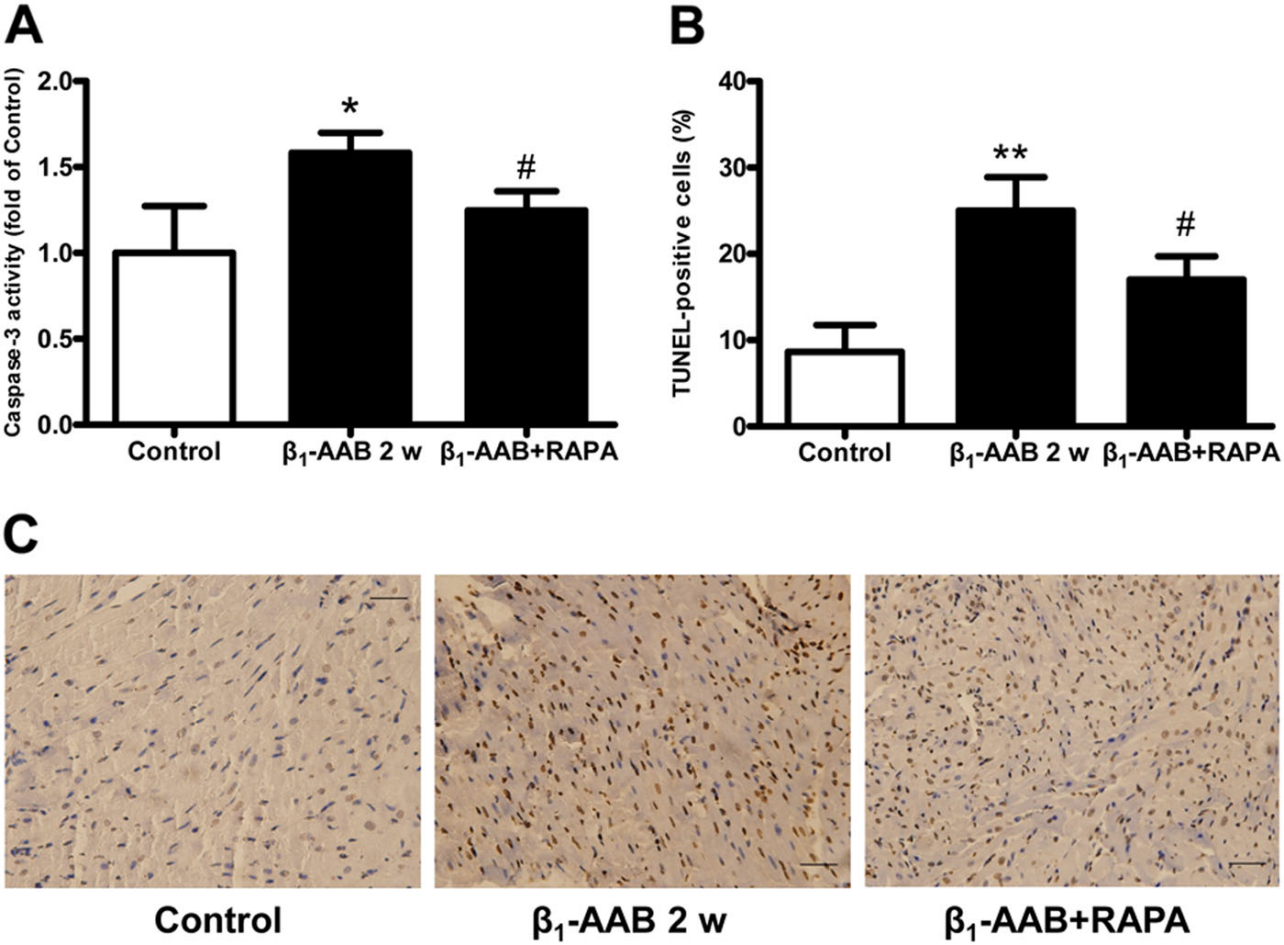
Cardiomyocyte apoptosis in actively immunized rats was reduced by upregulating autophagy with rapamycin (RAPA). **a** Effect of RAPA on caspase-3 activity in actively immunized rats 2 w after β_1_-AABs stimulation. **b** Quantification of TUNEL-positive cells from (**c**). **c** Representative TUNEL staining showing that TUNEL-positive cardiomyocytes were decreased after RAPA stimulation. Scale bar was 40 μm. Data are expressed as means ± SEM (*n* = 6 per group). *P < 0.05 vs. the control, **P < 0.01 vs. the control, and ^*#*^*P < 0.05* vs. the β_1_-AAB group

To investigate the effect of decreased myocardial autophagy induced by β_1_-AABs on myocardial apoptosis, RAPA (rapamyosin), an mTOR inhibitor, was used to upregulate myocardial autophagy. The results showed that increased autophagy could reduce caspase-3 activity and the number of TUNEL-positive cardiomyocytes of actively immunized rats, indicating that autophagy upregulation could effectively reverse cardiomyocyte apoptosis induced by β_1_-AABs (Fig. 3).

### β_1_-AABs induced apoptosis in neonatal rat cardiomyocytes

**Fig. 4:**
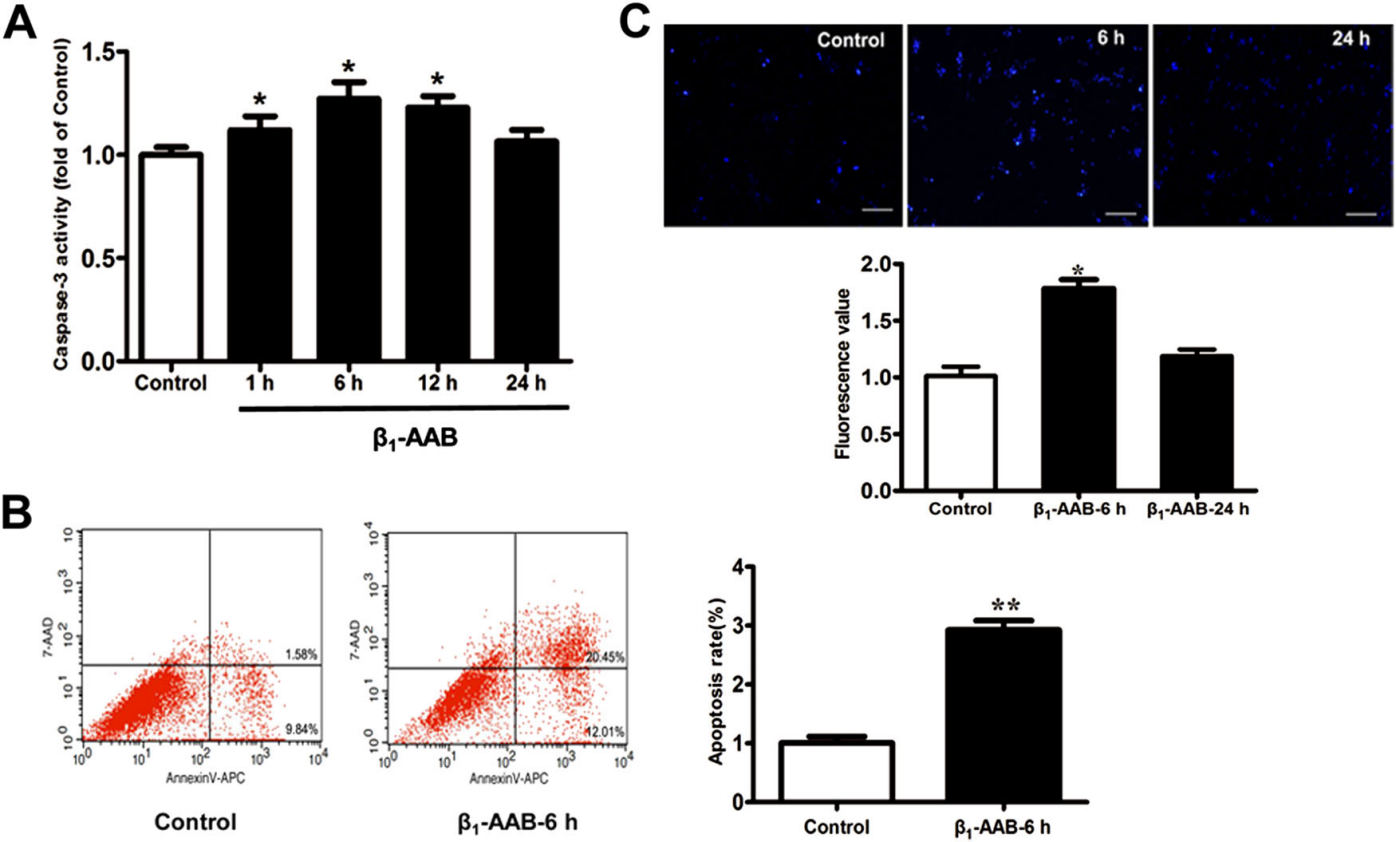
Change of β_1_-AAB-induced apoptosis in neonatal rat cardiomyocytes. **a** Activity of caspase-3 in neonatal rat cardiomyocytes after β_1_-AABs stimulation at 0, 1, 6, 12, and 24 h. **b** Data of Annexin V/7-AAD double staining flow cytometry. The percentage for each panel indicates the percentage of apoptotic cells. **c** Hoechst 33258 staining after β_1_-AABs stimulation at 6 and 24 h. Normal cells manifest as lighter blue-stained nuclei, while apoptotic nuclei produce dense, bright blue fluorescence. Scale bar was 100 μm. Data are presented as means ± SEM (*n* = 6 per group). *P < 0.05 vs. the control and **P < 0.01 vs. the control

It was found that caspase-3 activity in neonatal rat cardiomyocytes was significantly increased 1 h after β_1_-AABs treatment and it remained high for 12 h, and then returned to normal at 24 h (Fig. 4a). The data of Annexin V-APC/7-AAD double staining flow cytometry were consistent with the results mentioned above, in which after 6 h of β_1_-AABs stimulation, Annexin V-positive/7-AAD-negative staining cells and both Annexin V and 7-AAD-positive staining cells were all increased, indicating that β_1_-AABs could induce apoptosis in neonatal rat cardiomyocytes (Fig. 4b).

Further, Hoechst 33258 was used to stain neonatal rat myocardial cells. The results showed that without β_1_-AABs, blue staining of the nucleus was hypochromic. At 6 h after β_1_-AABs stimulation, bright blue nuclei appeared and the fluorescence intensity was significantly higher than in the control group, and at 24 h after β_1_-AABs stimulation, the fluorescence intensity had recovered (Fig. 4c). The above results showed that neonatal rat cardiomyocyte apoptosis increased with the presence of β_1_-AABs.

### Autophagic flux induced by β_1_-AABs increased early and then decreased in primary neonatal rat cardiomyocytes

**Fig. 5:**
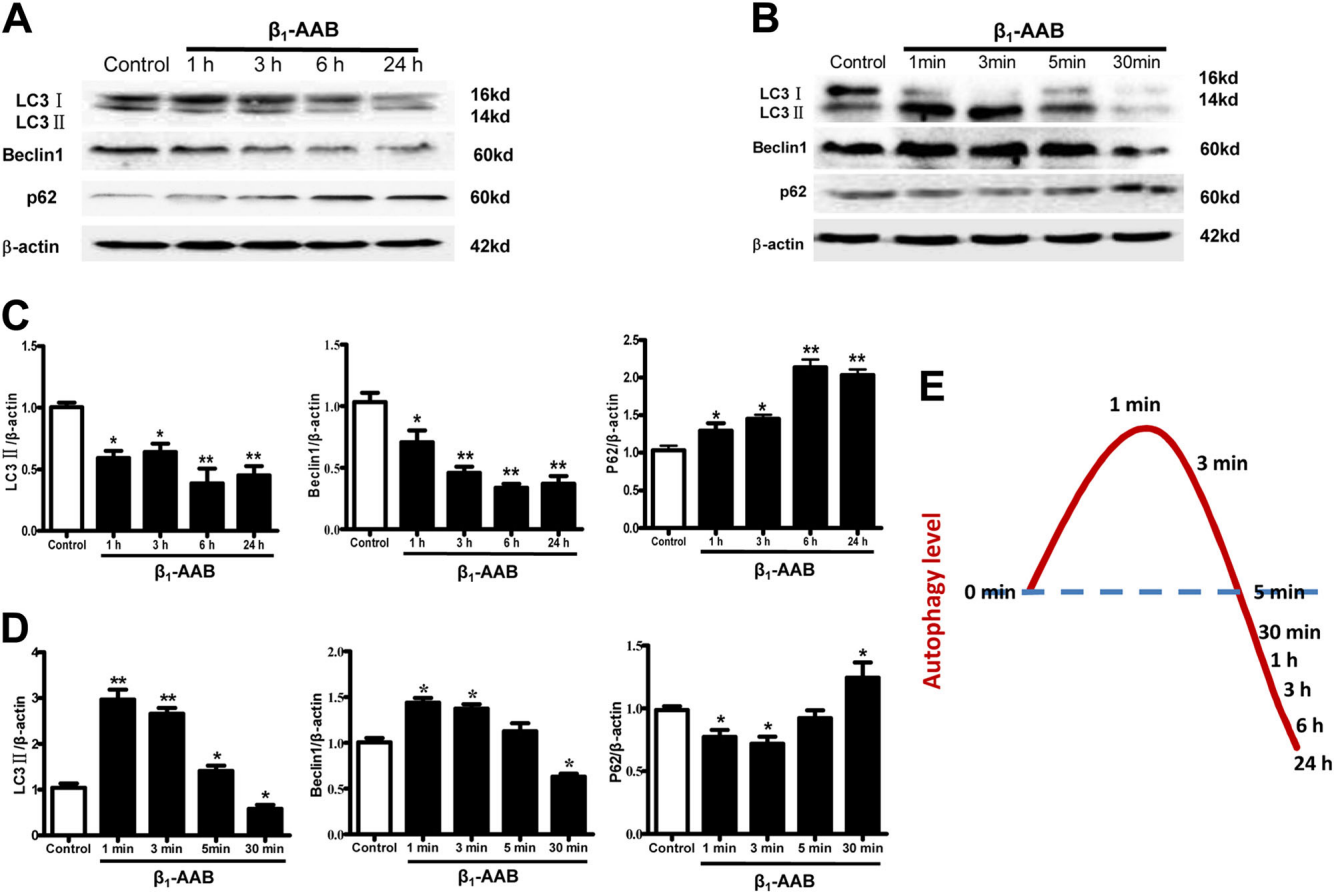
β_1_-AAB-induced autophagic flux increased early and then decreased in primary neonatal rat cardiomyocytes. **a** The protein expression of LC3, Beclin1, and p62 at 0, 1, 3, 6, and 24 h after β_1_-AABs stimulation. **b** Representative Western blot showing the protein expression of LC3, Beclin1, and p62 at 0, 1, 3, 5, and 30 min after β_1_-AABs stimulation. **c** Quantification of Western blot data from (**a**). **d** Quantification of Western blot data from (**b**). **e** Schematic representation of time course of autophagy level in primary neonatal rat cardiomyocytes after β_1_-AABs stimulation. Data are expressed as means ± SEM (*n* = 6 per group). *P < 0.05 vs. the control and **P < 0.01 vs. the control

The results showed that the protein levels of LC3 and Beclin1 in primary neonatal rat cardiomyocytes were significantly lower than in the control group at 1, 3, 6, and 24 h after β_1_-AABs stimulation, whereas p62 accumulated and was much higher than in the control group (Fig. 5a, c), suggesting that the myocardial autophagic flux was significantly decreased. To determine if the autophagy level induced by β_1_-AABs in primary neonatal rat cardiomyocytes would increase in an earlier stage, we stimulated the primary neonatal rat cardiomyocytes with β_1_-AABs at 1, 3, 5, and 30 min. The data indicated that the protein levels of LC3 and Beclin1 were significantly higher than in the control group and the p62 protein level decreased significantly in the primary neonatal rat cardiomyocytes at 1 and 3 min after β_1_-AABs stimulation. In contrast, the protein levels of Beclin1 and p62 had no significant difference compared with the control group at 5 min after β_1_-AABs stimulation. The protein levels of LC3 and Beclin1 decreased markedly at 30 min after β_1_-AABs stimulation, and the p62 protein level was increased (Fig. 5b, d). The above results showed that autophagic flux induced by β_1_-AABs ascended early and then declined in primary neonatal rat cardiomyocytes (Fig. 5e).

### The early increase of autophagy was an adaptive stress response, and the activation of β_1_-AR-PKA contributed to late decreased autophagy induced by β_1_-AABs

**Fig. 6:**
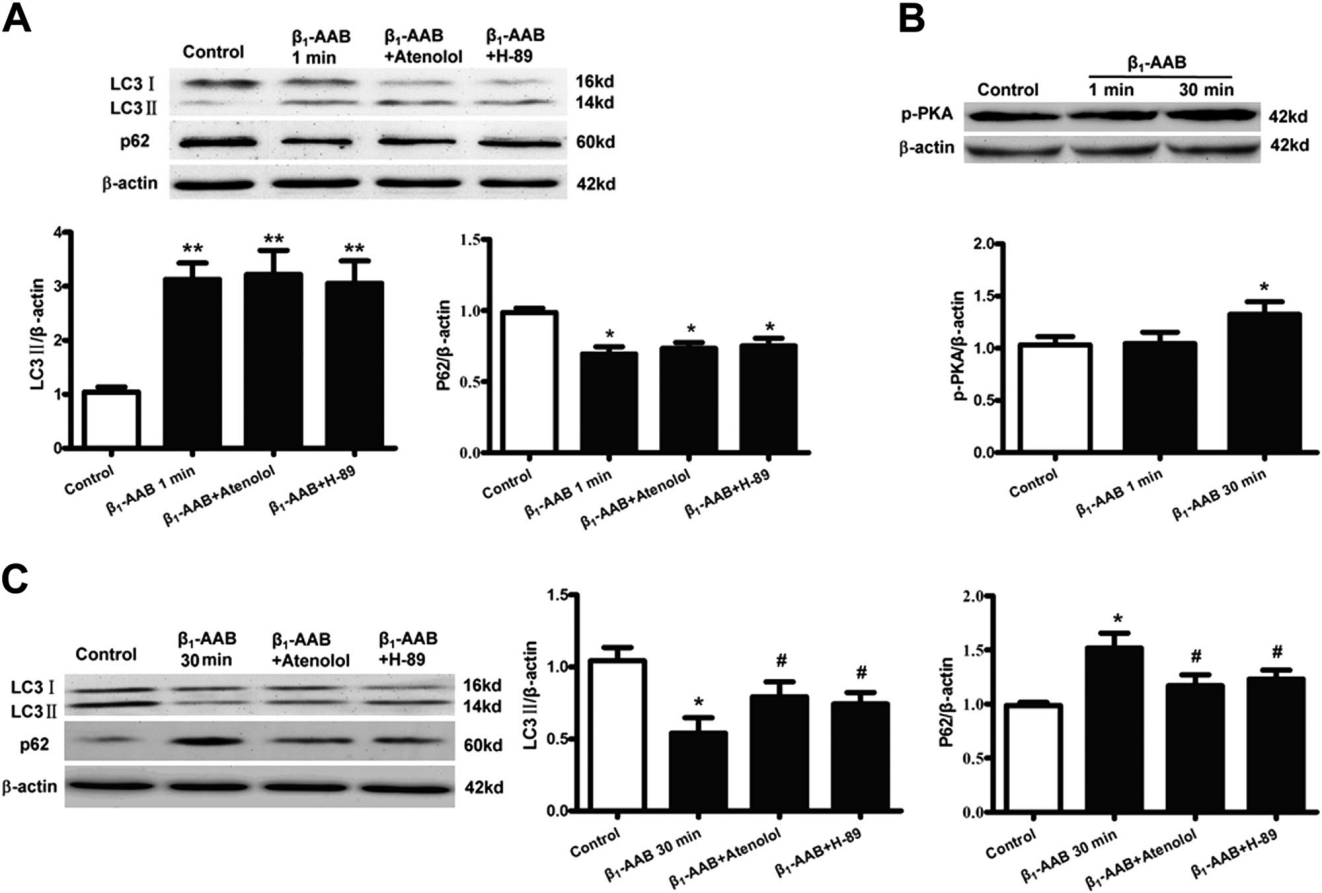
Effect of β_1_-AR and PKA on the changes of autophagy induced by β_1_-AABs. **a** Quantification by Western blotting and representative Western blots showing the protein expression of LC3 and p62 at 1 min after β_1_-AABs stimulation. **b** Quantification and representative Western blots showing the protein expression of p-PKA at 1 min and 30 min after β_1_-AABs stimulation. **c** Quantification by Western blotting and representative Western blots showing the protein expression of LC3 and p62 at 30 min after β_1_-AABs intervention. Data are expressed as means ± SEM (*n* = 6 per group). *P < 0.05 vs. the control, **P < 0.01 vs. the control, ^#^*P* < 0.05 vs. the β_1_-AAB group

To further confirm how the β_1_-AABs affect autophagy, atenolol, a selective β_1_-AR antagonist, was used to observe the role of β_1_-AR in autophagy induced by β_1_-AABs. In the present study, we found that autophagy was significantly increased at 1 min, and the autophagic flux was declined significantly at 30 min after β_1_-AABs intervention, so we chose 1 and 30 min to observe the possible mechanism of autophagic changes in the primary neonatal rat cardiomyocytes. The results showed that atenolol pretreatment had no significant effect on the increase of autophagy induced by β_1_-AABs at 1 min (Fig. 6a); however, pretreatment with atenolol could reverse decreased autophagy significantly at 30 min (Fig. 6c), indicating that the early increase of autophagy was an adaptive stress response that is possibly unrelated to β_1_-AR, and the activation of β_1_-AR contributed to late decreased autophagy. To further confirm these findings, protein kinase A (PKA), as an important signaling protein after β_1_-AR activation, was detected and the results showed that p-PKA had no change at 1 min and increased at 30 min after β_1_-AABs intervention (Fig. 6b). In an effort to elucidate the role of PKA in autophagy, we used the PKA inhibitor H-89 to treat the primary neonatal rat cardiomyocytes. Our data demonstrated that H-89 did not affect the level of autophagy at 1 min but recovered the decline of autophagy significantly at 30 min after β_1_-AABs stimulation (Fig. 6a, c), suggesting that PKA participated in the β_1_-AAB-induced reduction of autophagy. The above results showed that the early induction of autophagy by β_1_-AABs was an adaptive stress response, and the later declined autophagy induced by β_1_-AABs was due to β_1_-AR-PKA activation.

### Decreased autophagy participated in apoptosis of cardiomyocytes induced by β_1_-AABs

**Fig. 7:**
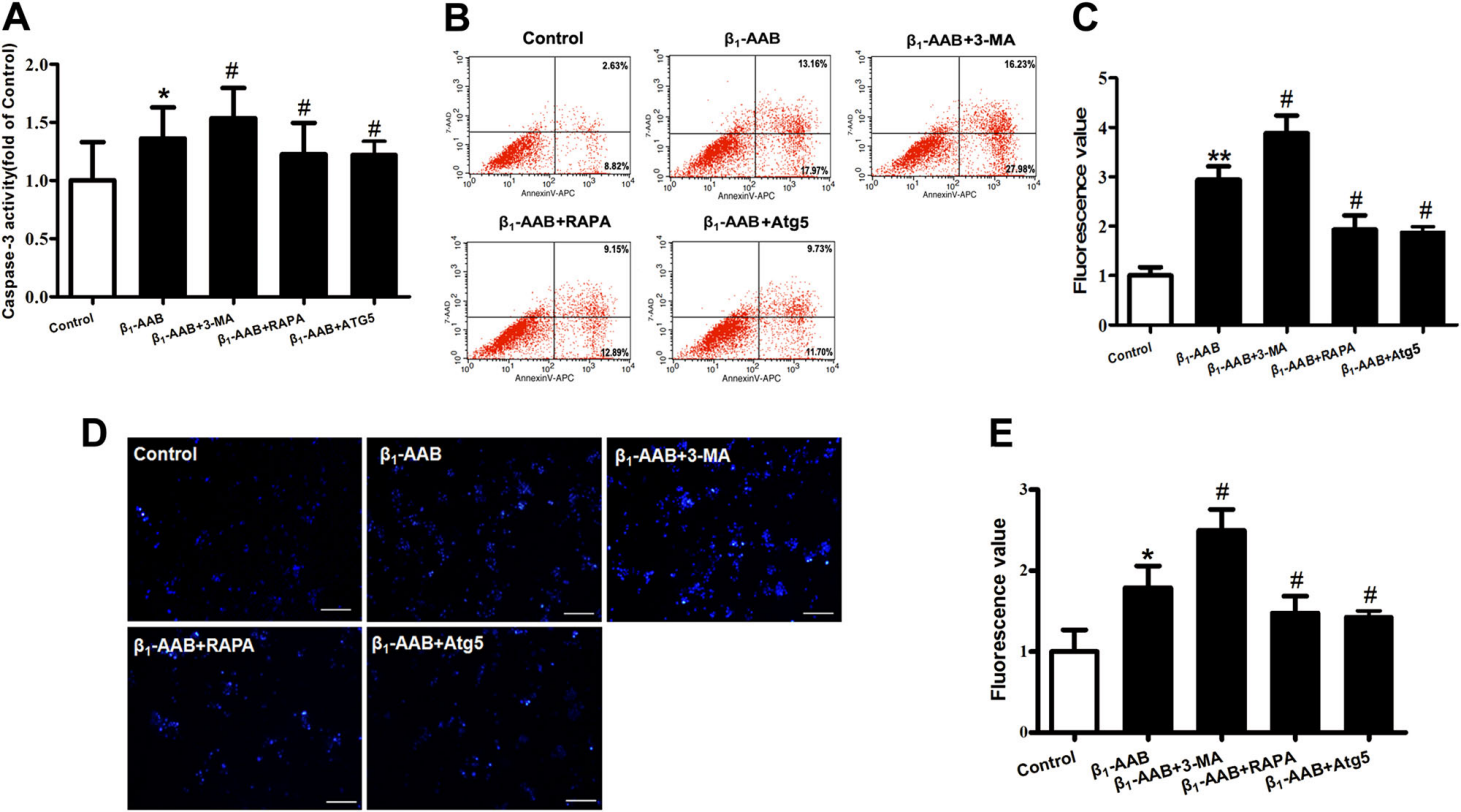
Effect of decreased autophagy on β_1_-AAB-induced cardiomyocyte apoptosis. **a** Caspase-3 activity in each group. **b** Representative flow cytometric assessment of apoptosis via Annexin V-APC and 7-AAD staining. **c** Flow cytometric quantification. **d** Representative images of neonatal rat cardiomyocytes stained with Hoechst 33258. Scale bar was 100 μm. **e** Quantification of Hoechst 33258 staining from (**d**). Data are presented as means ± SEM (*n* = 6 per group). *P < 0.05 vs. the control, **P < 0.01 vs. the control, ^#^*P* < 0.05 vs. the β_1_-AAB group

In our study, 3-MA and RAPA were used to restrain and improve autophagy in neonatal rat cardiomyocytes, respectively. The results showed that further increased caspase-3 activity was detected at 6 h after β_1_-AABs administration with 3-MA-pretreated cardiomyocytes, compared with the β_1_-AABs group, suggesting that inhibiting autophagy could upregulate apoptosis in myocardial cells. In addition, RAPA pretreatment reversed the effect of β_1_-AABs on caspase-3 activity at 6 h in the cardiomyocytes, indicating that upregulating autophagy could lead to the decline of apoptosis in myocardial cells (Fig. 7a). Similar results were found by Annexin V-APC/7-AAD staining, that is, the proportion of the right upper and lower quadrant cells was increased in the 3-MA-pretreated cardiomyocytes and recovered with RAPA-pretreated cells compared with those only treated with β_1_-AABs (Fig. 7b, c). Hoechst staining was conducted to prove the results and we found a stronger blue fluorescence in the 3-MA-pretreated myocardial cells compared with those only treated with β_1_-AABs, suggesting that 3-MA pretreatment could upregulate myocardial apoptosis (Fig. 7d, e). In contrast, blue fluorescence in RAPA-pretreated myocardial cells was weaker than in those only treated with β_1_-AABs, indicating that RAPA pretreatment may decrease myocardial apoptosis. To further confirm this idea, we added the Atg5 overexpression adenovirus expressing green fluorescent protein-infected neonatal rat cardiomyocytes to increase the level of autophagy. Atg5 as an autophagy-related protein is essential for autophagosome formation. The data showed that Atg5 overexpression could increase the level of autophagy (Supplementary Fig. S1) and reverse the effect of β_1_-AABs on apoptosis at 6 h in the cardiomyocytes (Fig. 7). All of the data showed that decreased autophagy participated in the apoptosis of cardiomyocytes induced by β_1_-AABs.

### Decreased autophagy promoted the β_1_-AAB-induced mitochondrial apoptosis pathway in cardiomyocytes

**Fig. 8:**
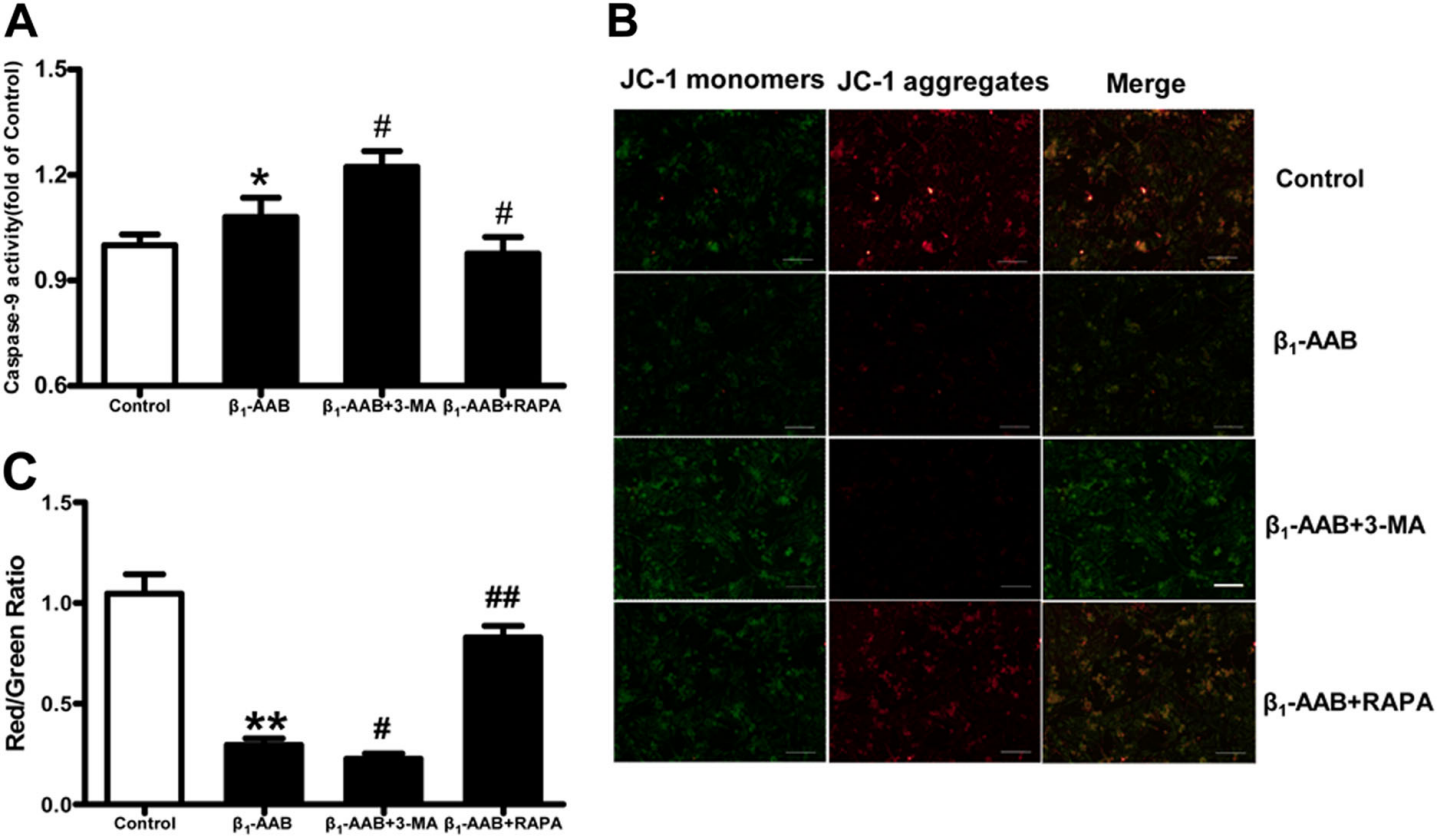
Effect of decreased autophagy on the β_1_-AAB-induced mitochondrial apoptosis pathway in cardiomyocytes. **a** Caspase-9 activity in primary rat neonatal cardiomyocytes in different groups. **b** Representative JC-1 staining of cardiomyocytes after β_1_-AAB stimulation in different groups. Scale bar was 100 μm. **c** Quantification of red/green fluorescence ratio from (**b**). Data are expressed as means ± SEM (*n* = 6 per group). *P < 0.05 vs. the control, **P < 0.01 vs. the control, ^*#*^*P < 0.05* vs. the β_1_-AAB group, and ^*##*^*P < *0.01 vs. the β_1_-AAB group

Studies have shown that the variation of caspase-9 activity and the red/green fluorescence ratio of JC-1 staining were closely related to the mitochondrial apoptotic pathway^10^. In this experiment, we observed that the caspase-9 activity in the primary rat neonatal cardiomyocytes increased significantly 6 h after β_1_-AABs stimulation (Fig. 8a). JC-1 staining indicated that the ratio of red/green fluorescence was decreased, suggesting that the mitochondrial membrane potential declined with the presence of β_1_-AABs (Fig. 8b, c). Then, 3-MA pretreatment led to increased caspase-9 activity and a decline in the red/green fluorescence ratio, indicating a further decrease in the mitochondrial membrane potential (Fig. 8). In contrast, RAPA pretreatment restored the decreased mitochondrial membrane potential and inhibited caspase-9 activity (Fig. 8). In conclusion, decreased autophagy contributes to the promotion of the β_1_-AAB-induced mitochondrial apoptosis pathway in cardiomyocytes.

## Discussion

In the present study, we observed that β_1_-AABs significantly increased apoptosis in the myocardial tissue of actively immunized rats and in cultured primary neonatal rat cardiomyocytes. We also found that the level of autophagic flux increased early and then decreased in the presence of β_1_-AABs. Additionally, autophagy decline promoted myocardial apoptosis effectively through the mitochondrial apoptotic pathway.

A large amount of clinical evidence has shown that high-titer β_1_-AABs can be detected in the serum of 40–60% of patients with cardiac dysfunction^3^. β_1_-AABs are autoantibodies against the second extracellular loop of β_1_-AR (β_1_-AR ECII) and accordingly perform agonist-like effects. One study indicated that β_1_-AABs could lead to an increased beating rate of cultured neonatal rat myocardial cells^11^. Left ventricular systolic and diastolic dysfunction could be observed in rats after 18 months of active immunization with β_1_-AR ECII^12^. In addition, the removal of β_1_-AABs in the blood of patients with cardiac dysfunction using an immunosorbent technique could markedly improve their cardiac function^13^.

Myocardial apoptosis is one of the major causes of cardiac dysfunction^14^. As a main pathway for cell death, apoptosis provides a relatively stable internal environment by eliminating redundant and damaged cells^15^. However, excessive apoptosis can cause tissue injury and functional defects^16^. It has been demonstrated that cardiomyocyte apoptosis aggravated cardiac insufficiency by participating in the remodeling of the left ventricle^17^. Studies have confirmed that β_1_-AABs led to the activation of the cAMP-dependent protein kinase signaling pathway, thereby increasing caspase-3 activity, resulting in cardiomyocyte apoptosis^18^. Therefore, cardiomyocyte apoptosis induced by β_1_-AABs is one of the major factors leading to cardiac dysfunction^19^. In this study, caspase-3 activity and the number of TUNEL-positive cells increased at 2 weeks and 4 weeks, suggesting that β_1_-AABs could increase myocardial apoptosis in actively immunized rats. However, it remains unclear how β_1_-AABs induce cardiomyocyte apoptosis.

Studies have shown that autophagy is involved in the occurrence of apoptosis^20^. Autophagy functions by degrading cytoplasmic components and recycling cellular materials via lysosomal pathways^21^, which is important for maintaining the homeostasis of the intracellular environment^22^. Thus far, some scholars believe that both of them are biological cell death processes that may be activated by a number of co-regulatory factors^23^. Autophagy is upstream of the apoptosis signaling pathway because it can inhibit apoptosis by degrading damaged proteins or DNA^5^. β_1_-AABs that interacted with β_1_-AR had an agonist-like effect^24^, and β_1_-AR could significantly affect autophagy^25^. Our preliminary studies found that the levels of autophagy in the myocardium were decreased markedly both in actively and passively immunized rats, and the autophagy decline was involved in cardiac dysfunction induced by β_1_-AABs^7, 26^. In the present study, we found that the level of autophagy increased significantly at 1 day and 1 week after active immunization with β_1_-AR-ECII. Thereafter, the autophagic flux of myocardial tissues started to decrease after 2 weeks of active immunization, and it appeared as the protein levels of LC3 and Beclin1 were significantly decreased. The p62 protein level had accumulated to high levels because it could not be degraded, and the decline of autophagic flux persisted for 4 weeks after active immunization. The above results suggested that the level of myocardial autophagy increased early but decreased later with the long-term presence of β_1_-AABs. In addition, we compared the time course between myocardial autophagy and apoptosis, and we discovered an interesting phenomenon that the level of autophagy increased significantly at 1 day and 1 week after being actively immunized by β_1_-AR-ECII. However, there were no significant changes in myocardial apoptosis during the same time period. At 2 and 4 weeks after being actively immunized by β_1_-AR-ECII, the autophagy level declined markedly, and at this point, the level of myocardial apoptosis increased significantly. These results suggested that the decrease of autophagy induced by β_1_-AABs may play an important role in cardiomyocyte apoptosis. In order to verify these problems, we used the mTOR inhibitor RAPA to upregulate the autophagy level, and we found that the upregulation of autophagy could attenuate the apoptosis effectively induced by β_1_-AABs. It was further proven that the autophagy decrease might be an important mechanism of β_1_-AAB-induced cardiomyocyte apoptosis.

In order to confirm the role of autophagy changes in β_1_-AAB-induced cardiomyocyte apoptosis, we purified IgG antibodies from the serum of actively immunized β_1_-AAB-positive rats to obtain β_1_-AABs that could directly act on cells. Rat neonatal cardiomyocytes were isolated and cultured. Specific markers of cardiomyocytes cTnI (red fluorescence) and α-actin (green fluorescence) were identified by immunofluorescence staining^27^. The results showed that the isolated cells were cardiomyocytes and suitable for further experimental studies (Supplementary Fig. S2). It was found that the β_1_-AABs could increase the beating frequency in primary neonatal rat cardiomyocytes, suggesting that purified β_1_-AABs possessed biologically active, that is, they had agonist-like effects (Supplementary Fig. S3). The CCK-8 assay showed that the survival rate of the primary neonatal rat cardiomyocytes decreased significantly after the administration of β_1_-AABs for 1 h, and the decline in survival rate lasted 48 h (Supplementary Fig. S4), suggesting that purified β_1_-AABs could lead to death of cardiomyocytes.

Next, we further examined the effect of β_1_-AABs on the apoptosis and autophagy levels in the primary neonatal rat cardiomyocytes. The results showed that the apoptosis increased significantly at 1 h after being stimulated by β_1_-AABs in the primary neonatal rat cardiomyocytes. Then, we found that autophagy was significantly increased at 1 min, and then it quickly decreased to normal at 5 min after β_1_-AABs intervention. Subsequently, the autophagic flux declined significantly at 30 min. To further explore the mechanisms of how the β_1_-AABs affect autophagy via the activation of β_1_-AR, a selective β_1_-AR antagonist atenolol was used to observe the role of β_1_-AR in autophagy induced by β_1_-AABs. In addition, protein kinase A (PKA) is an important signaling protein after β_1_-AR activation^28^ and it has been reported that PKA could inhibit autophagy by phosphorylating the Ser12 site of the autophagy-related protein LC3^29^. Phosphorylation of PKA converts the enzyme from an inactive to an active state^30^, so p-PKA was detected and PKA inhibitor H-89 was used to treat the primary neonatal rat cardiomyocytes. We chose 1 min (autophagy increased) and 30 min (autophagy declined) to observe the possible mechanism of autophagic changes in the primary neonatal rat cardiomyocytes. The results showed that atenolol or H-89 pretreatment had no significant effect on the increase of autophagy induced by β_1_-AABs at 1 min; however, pretreatment with atenolol or H-89 could reverse the decreased autophagy significantly at 30 min, indicating that the early increase of autophagy was an adaptive stress response that is possibly unrelated to β_1_-AR-PKA, and the activation of the β_1_-AR-PKA contributed to the late decreased autophagy. In addition, studies have shown that β_1_-AR activation could also induce an exchange protein directly activated by cAMP (Epac)^31^. Epac could promote cardiac autophagy during cardiomyocyte hypertrophy^25^ or inhibit autophagy induced by the toxin^32^. Therefore, Epac may also play a role in the change of autophagy induced by β_1_-AABs, but whether Epac inhibits autophagy or promotes autophagy to antagonize PKA needs further confirmation. In summary, we believed that the early induction of autophagy by β_1_-AABs was an adaptive stress response to protect cardiomyocytes and the later declined autophagy induced by β_1_-AABs was due to β_1_-AR-PKA activation. In fact, we have confirmed that both selective β_1_-AR antagonist atenolol and β_2_-AR blocker ICI118551 could significantly reverse the decline of autophagy induced by β_1_-AABs in cardiomyocytes (Supplementary Fig. S5). However, our recent results showed that β_1_-AABs did not bind to β_2_-AR directly^19^. Therefore, we hypothesized that β_1_-AABs could influence autophagy by affecting the interaction between β_1_-AR and β_2_-AR.

By comparing the time course of the changes in apoptosis and autophagy induced by β_1_-AABs in the primary neonatal rat cardiomyocytes, we also found that the apoptosis level was increased significantly at 1 h after β_1_-AABs intervention, and the autophagy levels decreased markedly at the same time, suggesting that the occurrence of apoptosis may be related to autophagy decline. To further confirm the effect of decreased autophagy induced by β_1_-AABs on cardiomyocyte apoptosis, we used the mTOR inhibitor RAPA or Atg5 overexpression adenovirus to upregulate autophagy, and we found that myocardial cell apoptosis had recovered significantly. While using 3-MA to inhibit autophagy, the level of myocardial cell apoptosis was increased significantly. We confirmed that the autophagy decline was involved in the β_1_-AAB-induced apoptosis in neonatal rat cardiomyocytes. However, it is unclear how autophagy affects apoptosis in myocardial cells.

Apoptosis is mediated mainly by the mitochondrial apoptosis pathway, the death receptor pathway, and the endoplasmic reticulum stress pathway^33^. Previous studies have shown that the activation of hypoxia-induced autophagy could eliminate damaged mitochondria, prevent the release of cytochrome c and the activation of caspase-9 and other factors, and inhibit apoptosis, thereby reducing cell death^8^, this suggests that autophagy could regulate apoptosis via the mitochondrial pathway. Our previous studies have shown that the mitochondrial membrane potential declined^26^, and the mitochondrial structure was abnormal with the long-term presence of β_1_-AABs^12^. However, whether decreased β_1_-AAB-induced cardiomyocyte autophagy leads to apoptosis via the mitochondrial pathway has yet to be determined. In the present study, we found that caspase-9 activity increased, and the mitochondrial membrane potential decreased markedly in the neonatal rat cardiomyocytes stimulated by β_1_-AABs. Then, inhibiting myocardial autophagy by 3-MA resulted in the enhancement of caspase-9 activity, and the mitochondrial membrane potential decreased further. In contrast, upregulating autophagy by RAPA reversed the activity of caspase-9 effectively, and the mitochondrial membrane potential was partially restored. Therefore, it can be concluded that autophagy may be involved in cardiomyocyte apoptosis induced by β_1_-AABs via the mitochondrial apoptotic pathway, which affects the death of cardiomyocytes and changes in heart function.

In conclusion, decreased cardiomyocyte autophagy induced by β_1_-AABs is crucial in the occurrence of cardiomyocyte apoptosis. We hypothesize that the reduction of apoptosis through the proper regulation of autophagy could decrease the loss of cardiomyocytes and improve heart function. This study offers new insights for the treatment of β_1_-AAB-positive patients with cardiac dysfunction.

### Perspective

Previous studies have shown that apoptosis is involved in the pathogenesis of various cardiovascular diseases; however, there are still have some problems with regulation of cell death by apoptosis inhibitors. Research have shown that defects in apoptosis underpinned both tumorigenesis and drug resistance^34^. Therefore, understanding how to regulate apoptosis properly could be the important step to treat these diseases. As studies have demonstrated, apoptosis could be affected by autophagy^35^. If the β-blockers are not suitable for some β_1_-AAB-positive patients with cardiac dysfunction^36^, then the regulation of apoptosis through autophagy would be a better way to reduce myocardial damage.

## Materials and methods

### Animals used in the study

Healthy male 8-week-old Wistar rats (140–160 g) were obtained from the Animal Center of Shanxi Medical University. The use and planning of the laboratory animals was approved by the Ethics Committee of Shanxi Medical University and we followed the People’s Republic of China’s Guidelines for the Care and Use of Laboratory Animals.

### Active immunization and rapamycin treatment

Animals were randomly divided into two groups: the β_1_-AR-ECII peptide immunized group and the control group. The β_1_-AR-ECII peptides were dissolved in Na_2_CO_3_ solution (100 mM, pH 11.0) to a final concentration of 1 mg/ml and then they were diluted in normal saline. The antigen solution, together with Freund’s complete adjuvant by an equal proportion, was emulsified and multiply-injected subcutaneously into the back of the rats (0.4 μg/g) during the first immunization. Booster immunizations were repeated every 2 weeks by a single subcutaneous injection, and the antigen was emulsified in Freund’s incomplete adjuvant. In the control group, the antigen solution was replaced with Na_2_CO_3_ solution. Rapamycin (RAPA) stock solution was prepared by dissolving rapamycin in DMSO (25 mg/ml) and storing it until it was diluted with PBS for intraperitoneal injection. Since the decrease of myocardial autophagy and the increase of myocardial apoptosis induced by β_1_-AABs occurs at 2 weeks after the active immunization, RAPA administration started 3 days before this decrease (day 12), beginning at 0.5 mg/kg/day in the first 3 days, and then it was adjusted to 0.25 mg/kg/day until the end of this study (4 weeks).

### Positive or negative serum IgGs were purified by affinity chromatography

The chromatographic column was placed at room temperature for 30 min. We mixed 0.5 ml serum with 0.5 ml binding buffer and then put the mixture in the affinity column. We washed the IgGs with 5 ml elution buffer (0.5 ml/min) and then we collected them in centrifuge tubes previously equipped with neutralizing buffer. The protein was quantified with a BCA kit (Thermo Scientific, 23228).

### Measurement of caspase-3 activity

In this study, a caspase-3 Activity Colorimetric Assay Kit (Nanjing Biobox Biotech. Co., Ltd., BA30100, Nanjing, China) was used to detect the caspase-3 activity to reflect the degree of apoptosis. First, 100 μl lysis buffer was added to lyse myocardial tissues and neonatal rat cardiac myocytes, then protein was measured with a BCA kit. Using a pipette, we placed 100 μg protein/50 μl volume in the administration group (using a pipette, we placed 50 μl lysate in the control group), and then 50 μl 2× reaction solution (we added 0.5 μl DTT/50 μl before using) and 5 μl Ac-DEVD-pNA was successively added into the administration and control groups, and incubated at 37 °C overnight. Finally, assay sample absorbances were measured at 450 nm. Caspase-3 activity was determined as follows: corrected fluorescence value = OD induced group/OD negative control group. The activity of the control group was defined as 1 to calculate the relative activity of caspase-3 of the other groups.

### TUNEL assay

An In Situ Cell Death Detection Kit, POD (peroxidase) (Roche Diagnostics) was used to detect the fragmentation of nuclear DNA in the early stages of apoptosis in the myocardial tissues. First, myocardial tissues were fixed with 4% paraformaldehyde, embedded in paraffin, and sliced. They were then dipped in xylene 2 times (5 min each time), and hydrated with an ethanol gradient (100, 90, 80, and 70% ethanol) each for 3 min. The tissues were treated with proteinase K for 15–30 min and washed twice with PBS. The TUNEL (terminal deoxynucleotidyl transferase dUTP nick end labeling) reaction mixture was prepared, added to each slice, and washed thrice with PBS. Then, 50 μl Converter-POD was added onto each specimen for 30 min and they were washed thrice with PBS. DAB (50–100 μl) was added onto the tissues and allowed to react for 15 min, and then the tissues were washed thrice with PBS, dyed by hematoxylin, and rinsed with tap water for a few seconds. Then, gradient alcohol dehydration and xylene transparent were performed. The apoptotic cells were observed under light microscope and photographed. Samples generated an insoluble brown substrate at the site of DNA fragmentation, while the normal nuclei were stained blue by hematoxylin.

### Western blotting

The protein expression levels of p62, LC3, and Beclin1 were determined by Western blot analysis. Myocardial tissues were removed at 0 day, 1 day, 1 week, 2 weeks, and 4 weeks after immunization and neonatal rat myocardial cells were harvested at different time points after stimulation with 1 µM β_1_-AABs, then the tissues and cells were immediately lysed (Beyotime, P0013). After standing on ice for 1 h, they were centrifugated, the supernatant protein was extracted, and prepared for quantitative analysis of protein with a BCA kit. The supernatant was analyzed by SDS-PAGE assay (the sample volume was 50 µg). After electrophoresis and transfer, the PVDF membranes (Whatman, 10485289) were blocked with 5% non-fat milk powder in TBST buffer, then incubated with anti-Beclin1 monoclonal antibodies (1:1000; Cell Signaling Tech, 3495), anti-LC3B monoclonal antibodies (1:1000; Sigma, L7543), anti-SQSTM1/p62 polyclonal antibodies (1:1000; Cell Signaling Tech, 5114), anti-phospho-PKA catalytic subunit (Thr-197), monoclonal antibodies (1:500; Cell Signaling Tech, 4781), and anti-β-actin monoclonal antibodies (1:1000; ZSGB-BIO; TA-09) at 4 °C overnight. The membranes were incubated with the corresponding secondary antibodies. Super ECL Plus (Applygen Technologies Inc., P1030) was added onto the membranes, which can be read by a camera’s automatic exposure system. Finally, the grayscale values of the straps were analyzed by Image J software, and the relative expression of the proteins was normalized on β-actin.

### Real-time PCR

The expression of *LC3* and *Beclin1* mRNA was measured by real-time PCR in myocardial tissues. First, approximately 0.5 μg of total RNA, which was isolated from myocardial tissues using RNAiso plus (TaKaRa, 500 μl per well), was reverse-transcribed into cDNA using the Prime Script RT Master Mix (TaKaRa). Then, we used SYBR Premix Ex TaqTM II (TaKaRa) to test the *LC3* and *Beclin1* mRNA expression. The primer sequences were as follows: LC3 (GenBank accession number, NM022867.2), sense: 5′-AGCTCTGAAGGCAACAGCAACA-3′ and antisense: 5′-GCTCCATGCAGGTAGCAGGAA-3′; *Beclin1* (GenBank accession number, NM001034117.1), sense: 5′-TTGGCCAATAAGATGGGTCTGAA-3′ and antisense: 5′-TGTCAGGGACTCCAGATACGAGTG-3′; and GAPDH (Gen-Bank accession number, NM_017008.3), sense: 5′-GGCACAGTCAAGGCTGAGAATG-3′ and antisense: 5′-ATGGTGGTGAAGACGCCAGTA-3′. The expression of *LC3* and *Beclin1* mRNA was standardized to GAPDH and data were quantified by the relative quantitative 2^−ΔΔCt^ method.

### Isolation, culture, and administration of neonatal rat cardiac myocytes

After disinfecting the area and administering anesthesia to the Wistar neonatal rats, we cut the sternums and removed the hearts, then put them into the cold PBS, rinsing 3–4 times. The ventricular tissue was cut into a 1 mm^3^ tissue block. We used mixed enzyme solution (0.25% trypsin + 0.0625% collagenase II) to digest the tissue block repeatedly. After filtration and centrifugation, the cells were seeded in 6-well plates for purification and then trypan blue stain was applied for 3 min. Cells were observed under a microscope and counted. At 36–48 h, the medium was changed for the first time, and after that, it was replaced every 2 days, with cardiac-specific markers α-actin and cardiac troponin I (cTnI) immunofluorescence staining performed to identify the cells 5 days later. As for the grouping and treatment of cells: the control group was treated with 1 μM negative IgG; we added 1 μM β_1_-AABs to the β_1_-AAB group; we added 10 mM 3-MA to the 3-MA pretreated group about 30 min before dosing with 1 μM β_1_-AABs; for the RAPA pretreated group, we needed to add 100 nM RAPA for 1 h and then we added 1 μM β_1_-AABs; for the group with ATG5 overexpression adenovirus infection, the primary neonatal rat cardiac myocytes were infected with adenovirus (HanBio, Shanghai, China) at a multiplicities of infection (MOI) of 60 for 4 h and the overexpression of ATG5 and LC3 were observed 24 h after infection.

### Annexin V-APC/7-AAD staining for apoptosis

Since Atg5 overexpression adenovirus (HanBio, Shanghai, China) was expressing green fluorescent protein, we selected APC Annexin V Apoptosis Detection Kit with 7-AAD to reflect the cell apoptosis. We used Annexin-V/7-AAD staining to distinguish the cells at different stages of apoptosis via flow cytometry. Neonatal rat cardiomyocytes were cultured in 6-well plates in DMEM medium with 10% FBS, then the medium was changed every 2 days. The cells were washed twice with cold PBS and resuspended at a concentration of 1 × 10^6^ cells/ml, then 5 μl Annexin V-APC and 5 μl 7-amino-actinomycin (7-AAD) were added to 500 μl of cell suspension (which was taken out from each sample), followed by incubation for 20 min at 37 °C in the dark. Finally, the cells were analyzed by flow cytometry for a cell count of 1 × 10^4^. Annexin V positive and 7-AAD negative is regarded as an indicator of early apoptotic cells and both Annexin V and 7-AAD positive as late stage apoptotic cells and necrotic cells. Our statistics are based on the proportion of cells in the right upper and lower quadrants of the graphs, accounting for the total number of cells to reflect the cell apoptosis.

### Hoechst staining

Hoechst 33258 is a blue fluorescence dye that can penetrate the cell membrane and exert low toxicity to cells so it can be used to determine cell apoptosis. Under a fluorescence microscope, normal cells showed with lighter blue nuclei, while apoptotic nuclei produced dense dyeing and bright blue fluorescence that can directly reflect the cell apoptosis. We determined the degree of apoptosis according to the fluorescence intensity analysis using the method that follows:^37^ myocardial cells of neonatal rats were inoculated in 6-well plates, and the medium was changed every 2 days. The cells were treated with 1 ml Hoechst dye after being washed twice with PBS, and then incubated for 30 min at 37 °C in a humidified, 5% CO_2_ environment. We then removed the cells, discarded the dye, washed the cells twice with PBS, and treated them with 1 ml PBS. Finally, we observed and took photos with an inverted fluorescence microscope (Olympus, IX51).

### Detection of caspase-9 activity

In our experiment, the mitochondrial pathway of apoptosis was reflected by the caspase-9 activity, which was detected by a caspase-9 assay kit (KeyGen Biotech Co. Ltd., GA402F). The experimental groups (30 μl of 100 μg protein) and the control group (30 μl PBS) were instilled with 50 μl 2× reaction buffer (pre-instilled with 0.5 μl DTT/50 μl), 10 μl ddH2O, and 10 μl caspase-9 substrate reaction solution for 1.5 h at 37 °C. Then, the fluorescence intensity in the different groups was determined by a fluorescence microplate reader (exciting wavelength = 485 nm, emission wavelength = 535 nm). To calculate the caspase-9 activity, the corrected fluorescence value = RFU (relative fluorescence unit) induced group/RFU negative contrast; the fluorescence value of the experimental groups was calculated based on the control group, which had a fluorescence value of 1.

### JC-1 staining

Mitochondrial membrane potential (ΔΨm) was monitored by JC-1, a lipophilic cationic dye that selectively enters the mitochondria. In healthy cells with normal ΔΨm, JC-1 spontaneously forms complexes known as J-aggregates with intense red fluorescence. In the case of mitochondrial membrane depolarization, the dye remains in its monomeric form with green fluorescence. The JC-1 red: green ratio has been used as a tool to estimate the changes in the ΔΨm. The detailed method follows: After removing the culture medium, the cells were rinsed twice with PBS and loaded with 1 ml of fresh medium and 1 ml of JC-1 staining for 20 min at 37 °C with 5% CO_2_, and the supernatant was removed. The cells were then washed twice with JC-1 staining (1×) and we added 2 ml of culture medium. We then observed and photographed the cells using laser scanning confocal microscopy.

### Statistical analysis

Data are expressed as means ± SEM. Statistical analysis was performed with SPSS software (version 16.0, SPSS Inc., Chicago, IL, USA). Two independent sample *t* tests were used to compare the means of two independent samples and one-way ANOVA was applied after a Bonferroni post hoc test for more than two samples. Significance was set at *P < *0.05.

## Electronic supplementary material

Supplementary information(DOC 29 kb)

Supplementary Figure S1(TIF 796 kb)

Supplementary Figure S2(TIF 2949 kb)

Supplementary Figure S3(TIF 2596 kb)

Supplementary Figure S4(TIF 2903 kb)

Supplementary Figure S5(TIF 1242 kb)
