# Supplementary material for: Decreased autophagy induced by β1-adrenoceptor autoantibodies contributes to cardiomyocyte apoptosis
Source: Cell Death Dis. 2018 Mar 14;9(3):406. doi: 10.1038/s41419-018-0445-9 (PMC5852148; doi:10.1038/s41419-018-0445-9)
Supplement: Supplementary file 1 — Supplementary information(DOC 29 kb) [file 41419_2018_445_MOESM1_ESM.doc]

**Supplementary methods**

1. Immunofluorescence staining

In this study, α-actin and cTnI were detected by immunofluorescence. Cells were seeded on 96-well plates at a density of 2 × 10^6^ cells/ml, and mouse anti-rat α-actin (1:100; Life Tech, 2775) and goat anti-rat cTnI (1:100; Abcam, ab47003) were added, and then after fixation, the 96-well plates were placed into the wet box for 1 night. After washing 3 times with PBS, donkey anti-mouse IgG (H+L) secondary antibody, Alexa Fluor 488 conjugate (Life Technologies, A21202), and Donkey anti-Goat IgG (H+L) Secondary Antibody Alexa Fluor 594 conjugate (Life Technologies, A11058) were added and they were incubated for 1 h. Finally, DAPI (Beyotime Biotech, C1005) was added to stain the nucleus and the cells were washed with PBS for 3 min. Laser scanning confocal microscope (Olympus, FV1000) was used to observe and take pictures of the cells.

1. Cell viability assay

The cell survival rate was measured by a cell counting kit-8 (CCK-8). Neonatal rat cardiomyocytes were cultured in 96-well culture plates at a density of 2 × 10^6^ cells/ml, and then the liquid was changed once every 2 days. After 1, 6, 12, 24, and 48 h of treatment with the reagents, 5 μl CCK-8 (Dojindo Molecular Technologies, CK04, Kumamoto, Japan) was added to the plates, and the cells were incubated for 90 min sequentially. Sample absorbance was measured at 405 nm with a microplate reader after the solution color changed to tan using the following equation: cell viability% = [(AS−AB)/(AC−AB)] × 100%, where AS is the absorbance of the samples, AC is the absorbance of the control, and AB is the absorbance of the DMEM media.

**Supplementary Figure Legends**

**Supplementary Figure S1** Atg5 overexpression could increase the level of autophagy. (A) Representative Western blot showing the protein expression of Atg5 and LC3 after Atg5 empty adenovirus (Ad-empty) and Atg5 overexpression adenovirus (Ad-Atg5) infection. (B,C) Quantification of Western blot data from (A). Data are expressed as means ± SEM (n = 6 per group). *P < 0.05 versus the control.

**Supplementary Figure S2** Immunofluorescence staining of cTnI (red fluorescence) and α-actin (green fluorescence) was performed to prove the cells that we isolated were rat neonatal cardiomyocytes. Scale bar was 100 μm.

Supplementary Figure S3 β_1_-AABs increased the beating frequency in primary neonatal rat cardiomyocytes 30 min after β_1_-AABs stimulation. (n = 6 per group). *P<0.05 versus the control.

**Supplementary Figure S4** Cell viability of primary neonatal rat cardiomyocytes declined obviously after administration of β_1_-AABs (n = 6 per group). *P<0.05 versus the control and **P<0.01 versus the control.

**Supplementary Figure S5** β_1_-AR and β_2_-AR contributed to the decline of autophagy induced by β_1_-AABs in H9c2 cardiomyocytes. (A) Representative Western blot showing the protein expression of LC3 and Beclin1 after 36 h of β_1_-AABs stimulation pretreated with/or without Atenolol. (B) Quantification of Western blot data from (A). (C) Representative Western blot showing the protein expression of LC3 and Beclin1 after 36 h of β_1_-AABs stimulation pretreated with/or without ICI118551. (D) Quantification of Western blot data from (C). Data are expressed as means ± SEM (n = 6 per group). **P<0.01 versus the control and ^#^P < 0.05 versus the β_1_-AAB group.
