# Supplementary figures and images for: Decreased autophagy induced by β1-adrenoceptor autoantibodies contributes to cardiomyocyte apoptosis
Source: Cell Death Dis. 2018 Mar 14;9(3):406. doi: 10.1038/s41419-018-0445-9 (PMC5852148; doi:10.1038/s41419-018-0445-9)

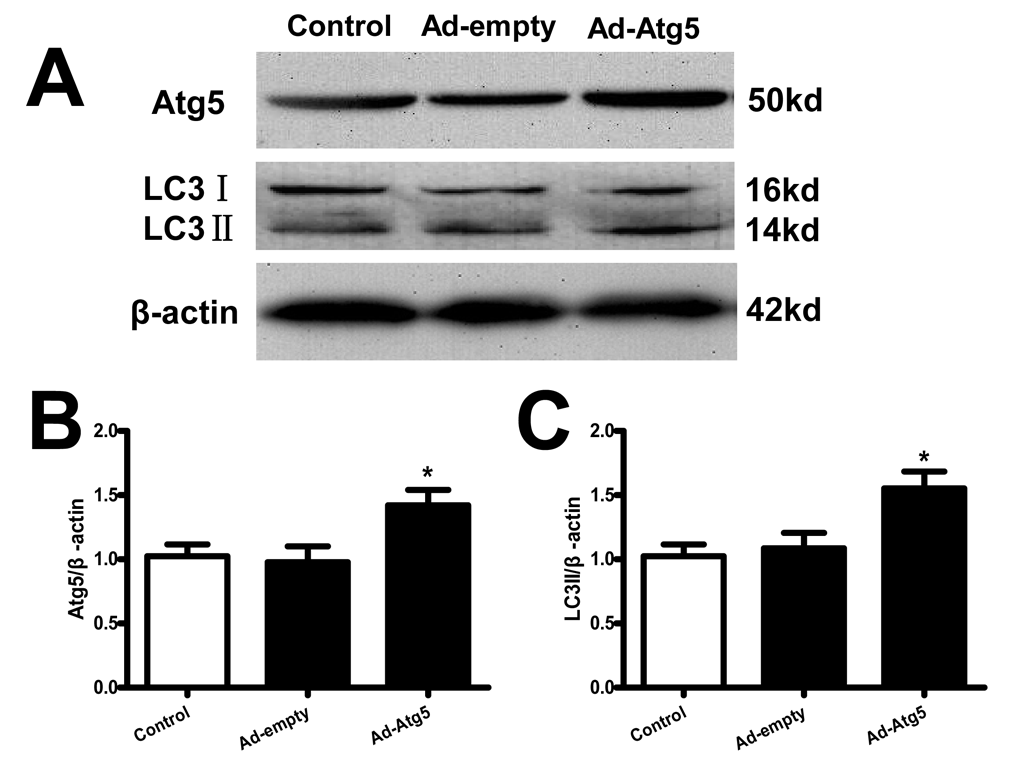

Supplement: Supplementary file 2 — Supplementary Figure S1(TIF 796 kb) [file 41419_2018_445_MOESM2_ESM.tif]

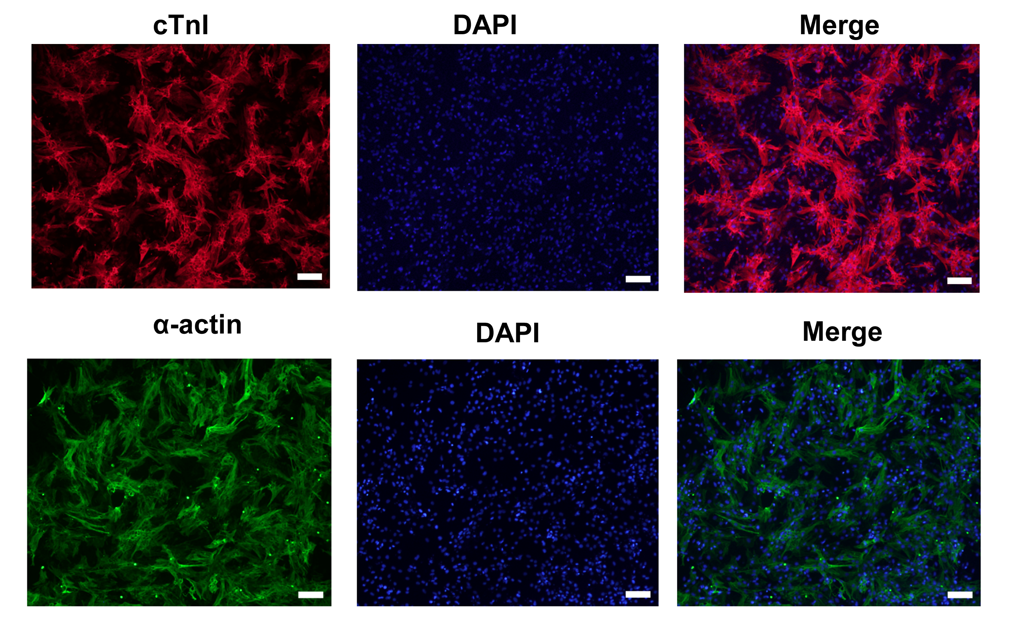

Supplement: Supplementary file 3 — Supplementary Figure S2(TIF 2949 kb) [file 41419_2018_445_MOESM3_ESM.tif]

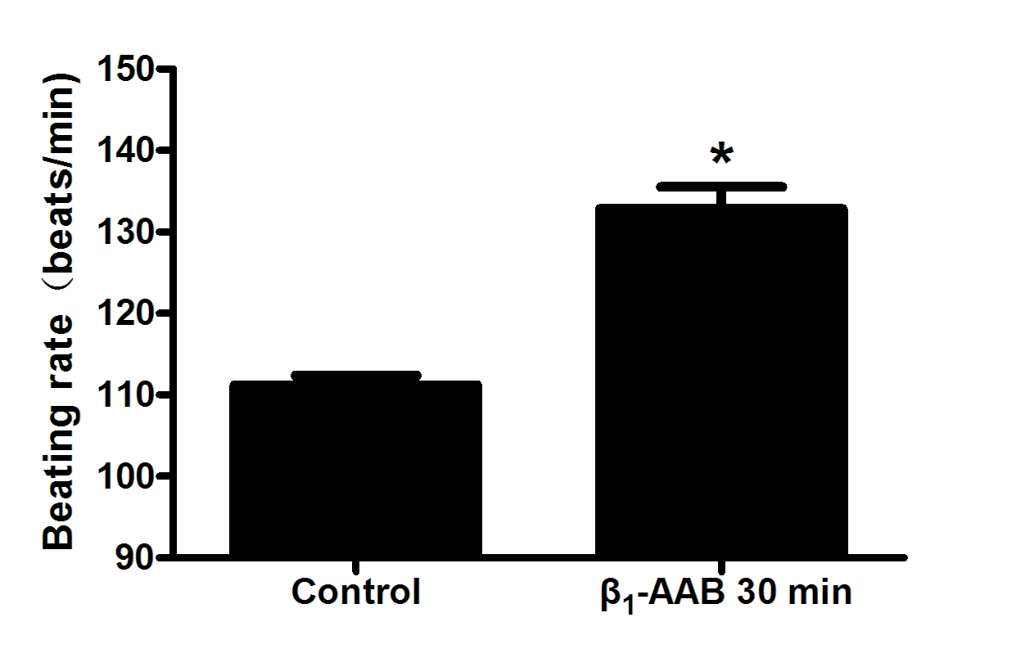

Supplement: Supplementary file 4 — Supplementary Figure S3(TIF 2596 kb) [file 41419_2018_445_MOESM4_ESM.tif]

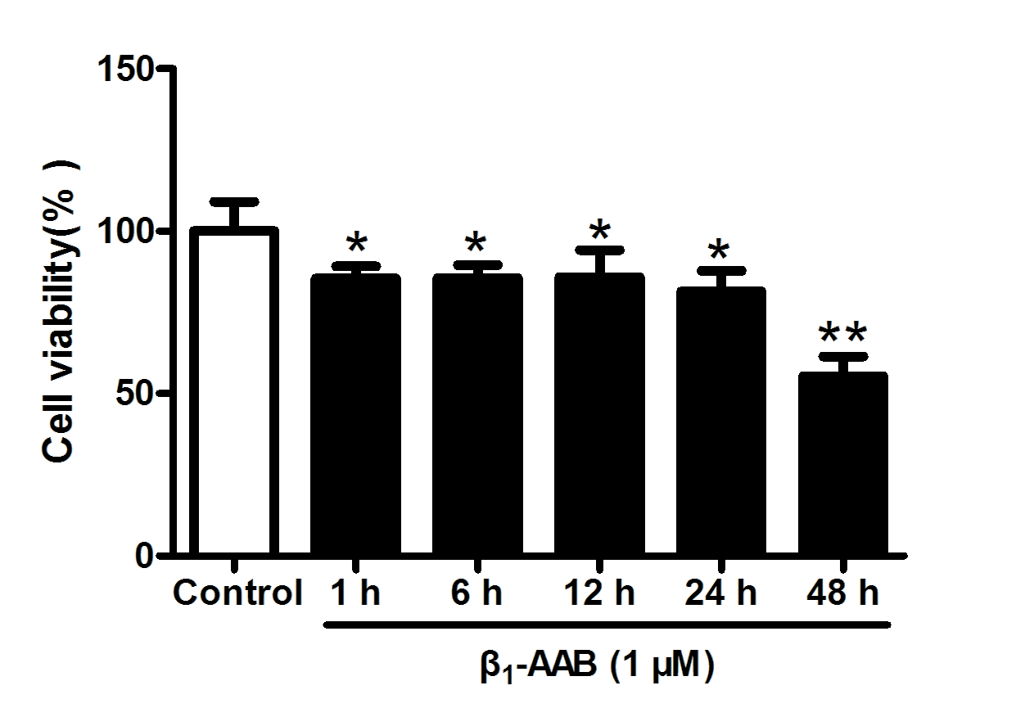

Supplement: Supplementary file 5 — Supplementary Figure S4(TIF 2903 kb) [file 41419_2018_445_MOESM5_ESM.tif]

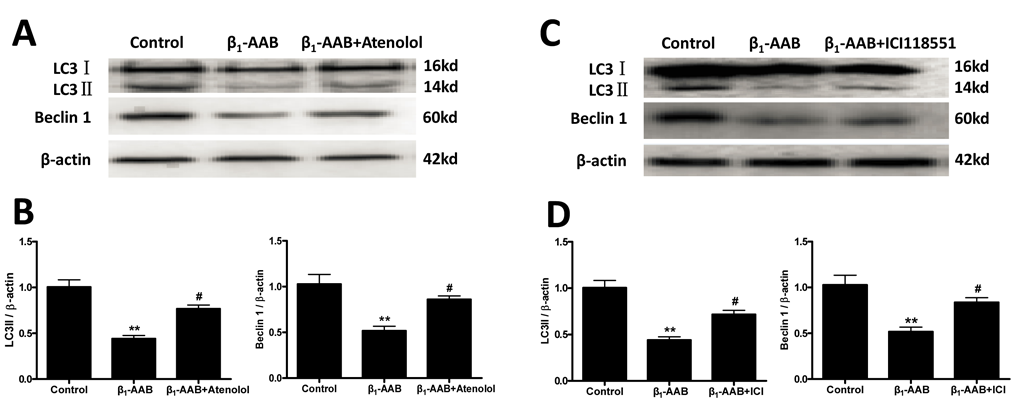

Supplement: Supplementary file 6 — Supplementary Figure S5(TIF 1242 kb) [file 41419_2018_445_MOESM6_ESM.tif]
